# Impact of tire debris on *in vitro *and *in vivo *systems

**DOI:** 10.1186/1743-8977-2-1

**Published:** 2005-03-24

**Authors:** Maurizio Gualtieri, Manuela Andrioletti, Paride Mantecca, Claudio Vismara, Marina Camatini

**Affiliations:** 1 Department of Environmental Science, University of Milano-Bicocca, P.zza della Scienza 1, 20126 Milano, Italy; 2 Department of Biology, University of Milano, Via Celoria, 26, Milano 20133, Italy

## Abstract

**Background:**

It is estimated that over 80% of respirable particulate matter (PM_10_) in cities comes from road transport and that tire and brake wear are responsible for the 3–7% emission of it. Data on the indicators of environmental impact of tire debris (TD), originated from the tire abrasion on roads, are extremely scarce, even though TD contains chemicals (zinc and organic compounds) which can be released in the environment.

**Methods:**

TD particle morphology was analysed with SEM, TEM and FIB instruments. TD eluates and TD organic extracts were tested at dilution series on human cell lines and *Xenopus laevis *embryos. 50 and 100 g/L TD were used for the eluates obtained after 24 h at pH 3 and the quantity of zinc present was measured with a ICP-AES. Eluates diluted to 1%, 10%, 50% in culture media and undiluted were used on *X. laevis *embryos in the FETAX test. HepG2 cells were exposed for 24 h to 0.05 – 50 μg/ml of zinc salt while A549 cells were exposed for 24, 48 and 72 h to 10, 50, 60, or 75 μg/ml of TD extract. *X. laevis *embryos were exposed to 50, 80, 100, or 120 μg/ml TD extract.

**Results:**

The solution of undiluted 50 g/L TD produced 80.2% mortality (p < 0.01) in *X. laevis *embryos and this toxic effect was three times greater than that produced by 100 g/L TD. Zn accumulation in HepG2 cells was evident after 4 h exposure. A549 cells exposed to TD organic extract for 72 h presented a modified morphology, a decrease in cell proliferation and an increase in DNA damage as shown by comet assay. The dose 80 μg/ml of TD extract produced 14.6% mortality in *X. laevis *embryos and 15.9% mortality at 120 μg/ml. Treatment with 80, 100, or 120 μg/ml TD organic extract increased from 14.8% to 37.8% malformed larvae percentages compared to 5.6% in the control.

**Conclusion:**

Since the amount of Zn leached from TD is related to pH, aggregation of particles and elution process, the quantity of TD present in the environment has to be taken into account. Moreover the atmospheric conditions, which may deeply influence the particle properties, have to be considered. The TD organic fraction was toxic for cells and organisms. Thus, because of its chemical components, TD may have a potential environmental impact and has to be further investigated.

## 1. Background

PM of urban areas is a complex mixture of organic and inorganic components [1].

Both fine (<2.5 μm) and ultrafine (<100 nm) particles and transition metal components are recognized to be responsible in determining toxicity and potential health effects [2,3]. The respiratory tract is the major site of exposure to PM, which may affect the tracheobronchial tree and the pulmonary alveoli. PM may cause cardiovascular and pulmonary complications and is responsible for increased percentage of lung cancer [4]. PM toxicity is demonstrated by epidemiological studies [2] and by investigations of laboratory mammals and pulmonary rodent cell lines [5,6]. Human cell lines were also utilised to study cytotoxicity, genotoxicity and induction of inflammation by soluble and insoluble compounds of PM [7-11]. PM_10 _[12,13], diesel exhaust particles (DEP) [14-17] and ultrafine particles [18,19] have received attention and been extensively analysed for their cytotoxicity, while the particulate deriving by tire abrasion on the road received a consideration limited to its impact on the soil, since it releases a large amount of zinc [20], and on the ambient air for its metal composition [21,22]. Previously, the presence of latex allergens in tire dust was suggested by Miguel et al. [23], who outlined that this component could be an important factor in producing latex allergies and asthma symptoms.

Tire debris (TD), generated from tire wear on roads, include particles with a size larger than 7 μm and range up to >100 μm, but also a population of smaller particles (<1 μm) [24], less than 20% of the total, according to Cadle and Williams [25]. More recently Fauser [26] examined the TD size distribution and concluded that it is a bimodal histogram with more than 90% by mass (in the range collected, i.e. <20 μm) smaller than 1 μm and the rest are larger than 7 μm. The total concentration of tire particles in a busy city road is in the range 1–10 μg/m^3^, with a mean value of 2.8 μg/m^3 ^which represents about 5% of the total airborne particles <20 μm. From these data and those of Tappe and Null [27] and Ntziachristos [28] it can be calculated that the 5–7% of these particles are in the PM_10 _size range of the respirable fraction.

Nelson et al. [29] and Evans [30] described the deleterious effect of whole tires and scrap on aquatic organisms produced by the release of zinc in the aquatic environment [31]. Moreover, Stephensen et al. [32] showed that the organic component is also toxic, since PAHs extracted from tire wear induced ethoxyresorufin-*O*-deethlyase (EROD) activity on fish.

The European Commission [33] has outlined the environmental risks related to the emission of TD during normal tire wearing, since the contribution of TD to B[a]P on respirable particles is slightly above 5% in comparison to DEP and will increase when diesel-associated emissions are reduced in the near future. Moreover, TD is a source of zinc to the environment [20,22,34].

The purpose of this paper is to investigate TD effects on the development of *X. laevis*, and on human cell lines. In previous studies, we described the shape [35-37], physical chemical characterization [38] of TD and preliminary results on their impact on a cell line [10] and living organisms [39]. Here, we present evidence that both TD eluates and TD organic extracts are highly toxic to living systems.

Michnowicz and Weaks [31], Evans [30] and Nelson et al.[29] identified zinc as the major source of eluate toxicity in the aquatic environment. Rubber compounds contain 2% zinc primarily as zinc oxide used as an activator in sulphur vulcanisation. Therefore, a certain quantity of zinc is leached from TD when it rains and when whole tires or scrap are used as water barriers. We analysed the effect of TD eluates on *X. laevis *embryos. The eluates were obtained by mixing a known quantity of TD in water at pH 3, since typical pH values of acid rain, caused by anthropogenic emissions, may be in the range of 3–5 [40]. Frog Embryo Teratogeneis Assay-*Xenopus *(FETAX) is a very sensitive aquatic model to assess toxicity and has been used. Moreover, the effect produced by a zinc salt added to the culture medium was evaluated on the human liver cell line HepG2.

TD extract, whose principal chemical compound was poly-isoprene, was tested on the A549 human alveolar cell line, a standardized model to study human lung damage [41] and on *X. laevis *embryos.

We present evidences that the toxicity of the eluates is in part related to the presence of Zn, as previously demonstrated by numerous authors on other living systems. Abernathy et al. [42] presented evidence that toxicity was not completely eliminated by the presence of the divalent chelator EDTA, and they suggested the presence of further toxic organic components. TD extract containing organic chemical compounds was toxic to the A549 human pulmonary cell line at a dose, which may correspond to the quantity present in the air humans inhale daily. Daily ventilator can be 20 m^3^, and contains 50 μg/m^3 ^PM_10 _in a polluted urban area. As previously reported 5–7% of PM is TD; thus the supposed quantity of TD inhaled may be 50–80 μg/m^3^/day.

## 2.Materials and methods

### 2.1 TD particle morphology

TD samples were obtained from laboratory degradation tests with a cryo-fracturing technique starting with tire scrap. The morphology of the fine 1–7 μm particles was analysed with a focused ion beam (FIB), and samples were prepared as described previously [37]. Particles were analysed with a scanning electron microscope (SEM) TESCAN and a transmission electron microscope (TEM) JEOL JEM 1220, according to the protocols described previously [38].

### 2.2 TD eluates

Eluates were obtained from 50 and 100 g TD placed in 1 L borosilicated glass bottles containing ultra pure water at pH 3. Zinc quantity was measured after 24 h with an Inductively Coupled Plasma-Atomic Emission Spectrometry (ICP-AES) instrument. A 206.200 nm emission wavelength was used to identify Zn ions which ensured that interfering species constituted a negligible part of the response signal. Zn (15.075 ppm, 5.05 ppm and blank) standards in Milli Q water at pH 3 were used for calibration. The bottles were placed in a mechanical shaker at 50 rpm for 24 h. Then different concentrations of the eluate at pH 3, which simulates the lowest pH value of acid rain caused by anthropogenic emissions [40], were prepared and sterilised. The dilutions were expressed as a percentage by volume. The TD undiluted leachate was defined as a 100% concentration, while the TD 10% leachate was diluted with 90% medium used in the FETAX test (1, 10, 50 and 100% eluate dilution series were used. Solutions were adjusted to the physiological pH of the FETAX medium before adding the salts.

### 2.3 TD organic extracts

Tire particles were placed in a cellulose extraction thimble and inserted in a Soxhlet assembly fitted with a 250 ml flask, and then 150 ml of dichloromethane was added. Particles were Soxhlet-extracted for 6 h and then either dried on a rotary vacuum evaporator or used for infrared spectroscopy analysis. TD extract was characterized by Fourier Transformed Infrared Spectroscopy (FTIR). Dichloromethane suspended extract was analysed. Briefly, 50 to 100 μl of TD extract were spread over KBr discs and left to dry. The KBr windows were analysed with a FTIR Midac M200 connected to a PC with a spectrum acquisition, storage and elaboration software. The pellet obtained after evaporation was suspended in DMSO at a ratio 10 μg/μl, frozen to -20°C, and used for the test on the A549 cell line and *X. laevis *embryos.

### 2.4 TD treatment on *in vitro *systems

The human lung A549 and the human liver HepG2 cell lines were routinely cultured at 37°C in Opti-MEM medium (Gibco, Life Technologies BRL, USA) supplemented with 10% heat inactivated foetal bovine serum (Gibco Life Technologies BRL, USA) and 1% antibiotics (penicillin\streptomycin) in a humidified atmosphere containing 5% CO_2_. The A549 cell line was used to test the effect of TD extracts, dissolved in culture medium to the concentrations 10, 50, 60 and 75 μg/ml for 24, 48 and 72 h. A positive control with DMSO at the concentration of 0.75% (v/v) was performed to verify the effect of the solvent alone at 75 μg/ml treatment.

The HepG2 cell line was used to evaluate the potential effect of the zinc salt ZnSO_4_7H_2_O. Cells, cultured as reported [43], were exposed for 2, 4 and 24 h to concentrations of Zn ranging from 0.05 to 50 μg/ml, dissolved in culture medium. The intracellular zinc level was directly measured by ICP-AES and expressed as ppm/10^6 ^cells.

#### 2.4.1 Cell viability

A549 cell proliferation and viability were evaluated with Trypan Blue (0,4%, Sigma). Cells were seeded in 6-well plates at 1 × 10^4 ^cells/well and treated as described in section 2.4. Control and treated samples were analysed with a reverse light microscope by adding 50 μl of Trypan Blue solution to the culture medium.

#### 2.4.2. Comet assay

Alkaline comet assay was performed on A549 cells [44,45]. Briefly, TD exposed cells were trypsinized and collected. 250 μl of 0.65% normal agarose was dropped onto frosted slides and left to gel. 100 μl of cell suspension was mixed 1:10 with 0.5% low-melting-point agarose, and 100 μl were pipetted onto the slides and allowed to gel; a final layer of 0.5% low-melting-point agarose was then added. The slides were immersed for 1 h at 4°C in the dark in cold lysing solution (2.5 M NaCl, 100 mM EDTA, 300 mM NaOH, 10 mM Tris, 34 mM N-lauroylsarcosine, pH 10); 10% DMSO and 1% Triton X-100 were added just before use. The slides were put in a electrophoresis chamber with 300 mM NaOH, 1 mM EDTA (pH 13.5) for 15 min. Electrophoresis was carried out at 0.8 V/cm for 15 min. The slides were rinsed three times with neutralization buffer (0.5 M Tris, pH 7.4) and stained with 20 μg/ml ethidium bromide. They were scored under a Zeiss Axioplan fluorescent microscope. For each dose, 400 cells/slide were scored and 25 individual comets were recorded for analysis with a self developed image system. The comet length percentage of DNA in the tail and the comet moment, defined as reported previously [45], were calculated.

#### 2.4.3 Transmission electron microscopy

Control and treated A549 cells, grown in 5.5 cm^2 ^plastic Petri dishes, were rinsed twice with 0.12 M phosphate buffer and fixed for 1 h with a solution containing 4% paraformaldehyde, 2% glutaraldehyde and 0.12 M sodium cacodylate buffer at pH 7.4. The cells were removed and centrifuged at 13.000 rpm for 15 min. The pellets were rinsed in 0.12 M sodium cacodylate buffer, and postfixed for 1 h in 1% osmium tetroxide buffered with 0.24 M sodium cacodylate. The samples were dehydrated and embedded in EPON 812 resin. Sections were stained with uranyl acetate and lead citrate and then examined with a Jeol JEM-1220 electron microscope.

### 2.5. TD treatment on *in vivo *systems

#### 2.5.1 Toxicity of TD eluates on *X. laevis*

Adult *X. laevis *(Centre d'élevage de *Xenopus *du CNRS, Rennes, France) were maintained in aquaria at 22 ± 2°C, alternating 12 h light/dark cycles, and fed a semi synthetic diet three times a week. For each bioassay, six females were injected with 900 IU of human chorionic gonadotropin (HCG). About 16 h later, these females were induced to lay eggs in a 140 mm plastic Petri dishes. The eggs were immediately artificially inseminated with a sperm suspension. One minute later, 30 ml FETAX solution [46] was added to each dish. Successful insemination was detected when all the eggs were oriented with the dark pole side up [47]. Eight hours later (stage 8, blastula) normal cleavage and development were ascertained. Test solutions were administered from stage 8 to stage 47 (free swimming larva), in a thermostatic chamber at 23 ± 0.5°C. The blastulae were exposed to 1, 10, 50, or 100% dilution series of TD eluates. For each female, three Petri dishes were used for the controls and each of the four solutions was tested in triplicate. At stage 47, all the larvae were evaluated for vitality. The surviving larvae were anaesthetized with MS 222 at the concentration of 100 μg/ml for morphological evaluation. For each experimental group, the number of dead embryos and surviving malformed larvae was recorded.

#### 2.5.2 Toxicity of TD organic extracts on *X. laevis*

*X. laevis *embryos were exposed to concentrations of 50, 80, 100 and 120 μg/ml TD organic extracts. Testing media were prepared diluting 10 μg/μl TD extract in FETAX solution, to reach the concentrations used.

The toxicity of extracts was examined by exposure of embryos for 120 h. At stage 8, 12 embryos were distributed in 40 mm Petri dishes containing 10 ml control or test solutions. Each experiment was repeated four times. Controls were in duplicate, as the first one was in control solution and the second one in control solution containing 1.2% (v/v) DMSO. This DMSO % produces 3–4% mortality when used in FETAX medium, according to Dresser et al. [48]. At the end of the experiments, the protocol reported in section 2.5.1 was followed.

### 2.6 Statistical analysis

The experiments with A549 and HepG2 cells were in triplicates and repeated at least three times. A ANOVA model and Kruskall-Wallis test (Statgraphics Plus 5.0 software) were run to detect statistical significant differences between the control and treated sample groups.

The relationship between the TD extract concentration and the percentage of dead or malformed larvae was investigate by the χ^2^-test and ANOVA model.

## 3. Results

### 3.1 Particle morphology

**Figure 1:**
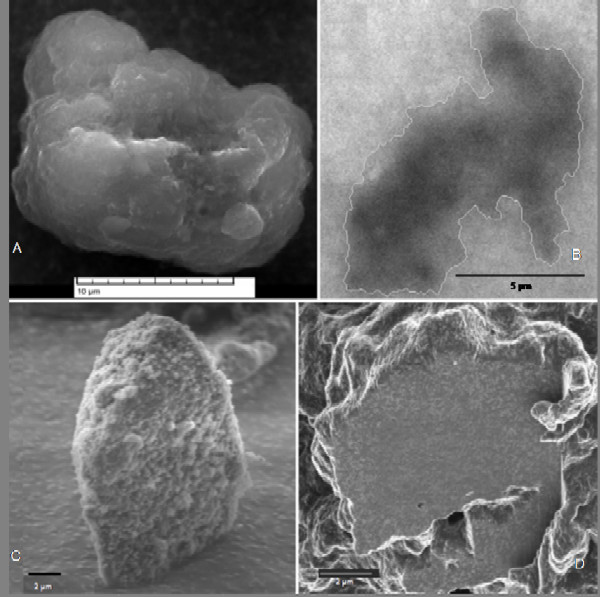
**Ultrastructural morphology of TD particles. **The morphology of TD particles is shown. A = a SEM image; B = a TEM image; C = a particle viewed with a focused ion beam instrument (FIB); D = FIB inside view of a TD particle.

Particle morphology is shown in Figure 1. The images were obtained respectively with SEM, (1,A), TEM (1,B) and FIB (1,C and D) instruments. SEM, TEM and FIB images illustrate the TD external roughness. The FIB technique revealed the particle shape modification after the laboratory leaching, as reported by Milani et al. [37]. Moreover, this technique visualises the internal particle morphology, since the high resolution image is coupled to an *in situ *sectioning, which shows variable density of the components (Fig. 1D). This aspect is under further investigation. The particle morphology, illustrated in Figure 1 and obtained as described in section 2.1, is very similar to the environmental collected tire particles [38] even if the external shape is not as rough as the ambient collected particles. Cryo-obtained particles have the same chemical composition of tire wear particles.

### 3.2 Chemical characterization

**Figure 2:**
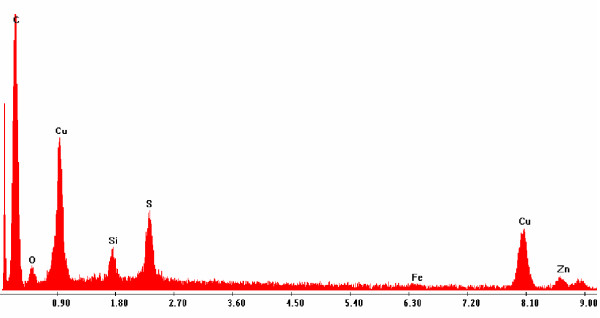
**EDX Spectrum of TD particle. **Microanalysis of a TD particle: a typical fingerprint of tire rubber is shown. This spectrum refers to the particle shown in Fig. 1A. The co-presence of Zn, S and Si is a significant marker to identify TD. Cu derives from the Cu 300-mesh grid used as support for TD particles.

**Figure 3:**
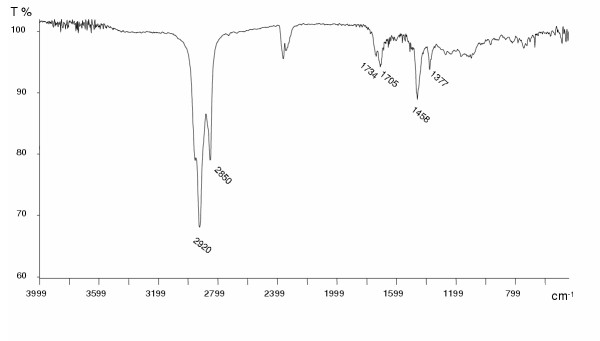
**FTIR Spectrum of TD extract. **Soxhlet extracted TD organic compounds analysed by FTIR spectroscopy. The poly-isoprene fingerprint is evident, which confirmed the presence of this rubber compound.

Identified by SEM morphology, rubber particles were analysed with EDX to determine elemental composition. Figure 2 presents the typical fingerprint of vulcanized tire compounds for the detection of S, Zn and Si, which are good TD environmental markers [38]. This EDX spectrum refers to the SEM image of Figure 1A. The Cu peak of the EDX spectrum derives from the material (Cu) which constitutes the grids on which TD particles were collected. In Figure 3, a FTIR spectrum presents peaks related to isoprene polymers. The most defined ones at 2920 and 2850 cm^-1 ^refer to CH_2 _stretching (symmetric and asymmetric), typical in isoprene polymer. The peaks at 2363 and 2334 cm^-1 ^refer to CO_2 _present in the apparatus, the 1734 cm^-1 ^peak relates to C = O stretching while 1705 cm^-1 ^refers to a CH_3 _group. Weaker peaks at 1458 and 1377 cm^-1 ^refer respectively to CH_2 _and CH_3 _groups.

### 3.3. In vitro systems

#### 3.3.1 Zinc salt on the HepG2 cell line

**Figure 4:**
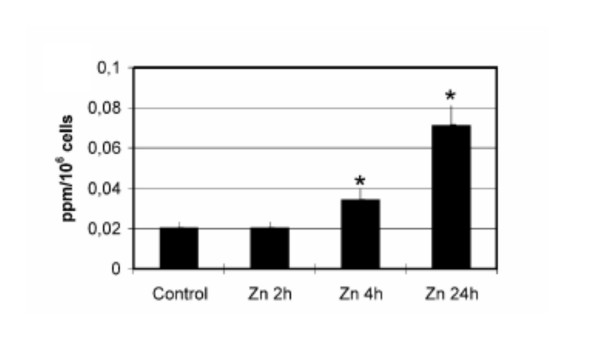
**Accumulation of Zn in HepG2 cells. **Zn levels in HepG2 cells exposed to 50 μg/ml ZnS0_4 _7H_2_0 for the time indicated and measured by ICP-AES. Data (ppm/10^6 ^cells) represent the mean of at least three replica with the standard deviation. *Significantly different from the control with the Kruskall-Wallis test (p ≤ 0.05).

ICP-AES analysis evidenced a time-dependent increase of intracellular Zn in HepG2 cells treated for 2, 4 and 24 h with 0.05 – 50 μg/ml. After 2 h exposure to 50 μg/ml Zn SO_4 _7H_2_0, Zn intracellular levels were comparable to those of controls. A significant accumulation of Zn (Kruskall-Wallis test, P < 0.05) was apparent after 4 and 24 h treatment (Fig. 4). The dose of 50 μg/ml was comparable to the highest Zn quantity leached by 50 g/L TD after 24 h at pH3.

#### 3.3.2 TD extracts on the A549 cell line

##### Cell viability and proliferation

**Figure 5:**
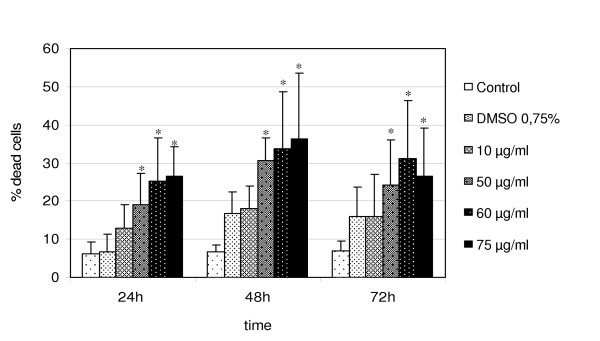
**A549 cell viability. **Percentage of dead A549 cells (evaluated by Trypan Blue exclusion method). Data refer to the mean and standard deviation of at least three independent experiments. The statistically significant differences (p ≤ 0.05) in viable cell number are marked (*).

**Figure 6:**
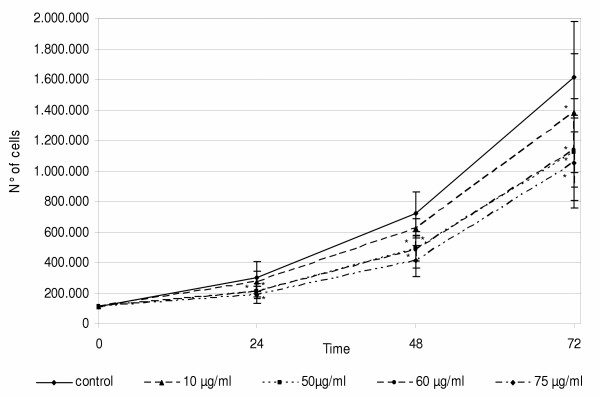
**A549 cell proliferation. **Cell growth curves at 24, 48 and 72 h after TD organic extract treatment at the doses indicated. A decrease of cell proliferation capability is evident, and it is time and dose dependent. Differences between control and treated samples are marked with * (p ≤ 0.05).

Cell viability and proliferation capability were evaluated. Viability was performed with the Trypan Blue exclusion test. An increase in cell death was apparent at 24, 48 and 72 h of exposure to the higher doses (50, 60 and 75 μg/ml) of TD extract in comparison to controls and DMSO-controls. The dose 10 μg/ml produced similar % of dead cells of DMSO-control (Fig. 5). The number of dead cells at 75 μg/ml at 72 h was lower and this result has to be further investigated. Cell proliferation decreases in time- and dose-dependent manner (Fig. 6). A significant decrease in cell proliferation was evident at 24 h and became more evident at 48 and 72 h.

##### DNA damage

**Figure 7:**
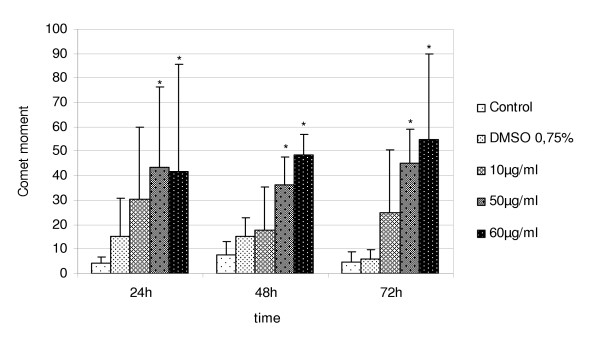
**DNA evaluation by comet assay of A549 cells. **DNA strand breakage in A549 cells exposed to TD organic extracts. Strand break formation was quantified with comet moment on control and treated samples. Data are mean ± S.D. of three independent experiments and show a dose-dependent increase of comet moment. An * shows significantly different data (p ≤ 0.05)

Single cell gel electrophoresis was performed to analyse DNA damage after the exposure of A549 cells to TD organic extract. Results are shown in Figure 7, where an increase in DNA strand breakage is evident in the treated sample group. The tail and comet moments are the most reliable quantitative indicators of DNA damage, since they refer to the distance of migration and the amount of DNA that has migrated from the head. The comet moment increases steeply as a function of the dose when 50 and 60 μg/ml are used. Differences among control, DMSO-control and exposed groups are shown in Figure 7.

##### Ultrastructure

**Figure 8:**
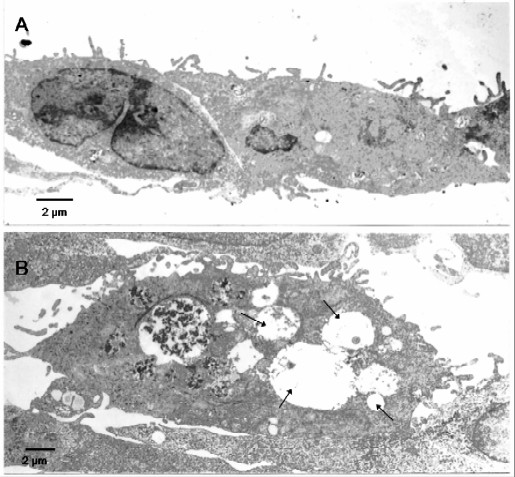
**Transmission electron microscope of A549 cells. **A = control cells present numerous microvilli and the normal cytoplasmic inclusions. B = a vacuolised cytoplasm (arrows) after exposition to TD organic extract. This modified morphology of the cytoplasm is frequent in cells treated with 75 μg/ml TD organic extract at 72 h. (bar = 2 μm).

At 72 h, control A549 cells showed typical type II pulmonary cell morphology with a flattened profile. Mitochondria, rough and smooth endoplasmic reticulum, Golgi apparatus and lamellar bodies were present; plasma membranes showed numerous microvilli (Fig. 8A). Cells exposed to the TD organic extracts presented modified morphology and the modifications were more evident at 72 h of treatment. The highest dose of TD organic extract produced a visible vacuolisation in the cytoplasm (Fig. 8B) as well as apoptotic nuclear images. These structural alterations were present in 50% of the cells at 72 h and 75 μg/ml dose.

### 3.4 *In vivo *systems

#### 3.4.1 TD eluates on *X. laevis *development

**Figure 9:**
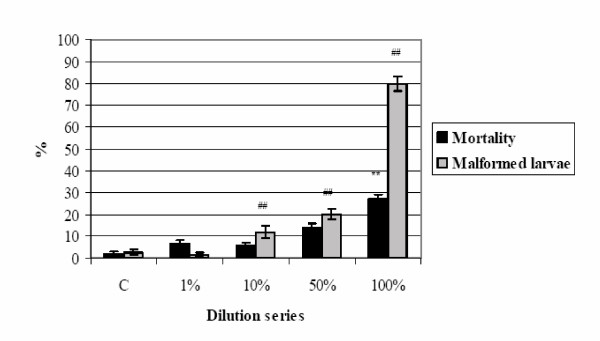
**50 g/L TD eluates and FETAX test. **Embryotoxic effects of 50 g/L TD on *X. laevis *embryos, 120 h, stage 47. (** p ≤ 0.01, % mortality compared to control, χ^2^-test; ## p ≤ 0.01, % malformed larvae compared to control, χ^2^-test).

**Figure 10:**
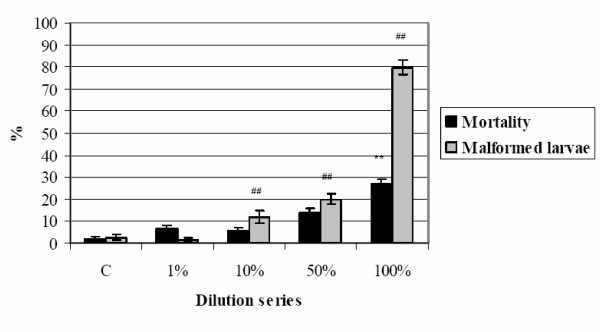
**100 g/L TD eluates and FETAX test. **Embryotoxic effects of 100 g/L TD on *X. laevis *embryos, 120 h, stage 47. (** p ≤ 0.01, % mortality compared to control, χ^2^-test; ## p ≤ 0.01, % malformed larvae compared to control, χ^2^-test).

After 24 h at pH 3, Zn concentration in 50 g/L TD was 44.73 μg/ml and in 100 g /L TD the quantity was 35.28 μg/ml. The effects of 50 and 100 g/L TD are presented in Figures 9 and 10. The 1% to 50% dilution series were not lethal; 100% 50 g/L TD produced a mortality percentage of 80.2% (p < 0.01) and 100% 100 g/L TD 26.8% (p < 0.01). Thus, the toxic effect of 50 g/L TD (Figure 9) was three times greater than that produced by 100 g/L (Figure 10), while the same solutions exhibited similar increases in malformation %. There was a significant difference between the control and exposed groups starting from 1% for 50 g/L TD (p < 0.01) and from 10% for 100 g/L TD (p < 0.01). Probit analysis of malformed larva percentages showed a TC_50 _of 40.2% for 50 g/L TD and 73.3% for 100 g/L TD. Therefore, eluates produced with 50 and 100 g/L TD had teratogenic effects. Zinc chloride embryotoxicity and teratogenicity were tested by FETAX and LC_50 _was reported to be 55 μg/ml for Zn and EC_50 _2.6 μg/ml, respectively [49].

#### 3.4.2 TD extracts on *X. laevis *development

**Figure 11:**
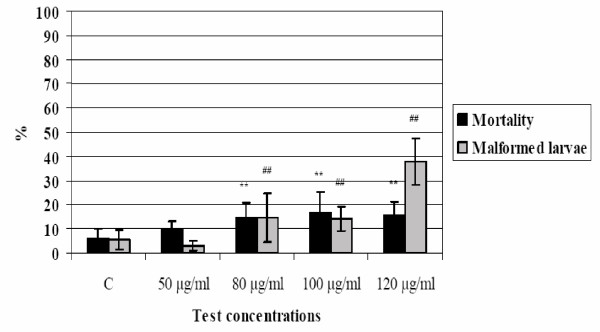
**TD organic extracts on *X. laevis *development. **Trend of mortality and malformed larvae percentage values after *X. laevis *embryos exposition to TD extracts. (** p ≤ 0.01, % mortality compared to control, χ^2^-test; ## p ≤ 0.01, % malformed larvae compared to control, χ^2^-test).

The effects produced by TD organic extracts are presented in Figure 11. The difference on mortality percentages in *X. laevis *larvae was highly significant (p < 0.01) between control (6.2 %) and exposed groups starting from 80 μg/ml concentration. At 120 μg/ml the percentage of mortality was 15.9%.

An increase of malformed larvae percentages was found at 80 and 100 μg/ml (14.8 %; 14.3 %; p < 0.01) compared to the control (5.6%). At concentration 120 μg/ml 37.8% of larvae were malformed. The most frequent malformation was the abnormal gut coiling, often coupled with generalized edema and/or microphthalmia. Some larvae were affected by heavy morphological alterations and so classified as monstrosity.

## 4. Discussion

The distribution of TD particles should be better analysed together with their resuspension properties once deposited on the land surface. At present TD size distribution [25,26,38] and chemical composition [20,26,38,50,51] have received sufficient attention to state that they can have an environmental impact. Actually, TD particles can be identified by inorganic [26,38,52] and organic [53] tracer species. A better understanding of their morphology has recently been obtained using a FIB instrument [37], which gives further information on the distribution of tire chemical compounds. It has been proven that both organic components [42,54,55] as well as inorganic ones [21,29,56] affect the environment. In the literature, it is reported that tire-tread formulations historically include about 2.5% Zn [57]. For several decades, tire wear materials have been recognized as a source of Zn in the environment [20,22,29,30], and recently it has been reported [20] that 10–40% of Zn from soil-applied TD was released in a labile pool. Thus, particular attention has to be devoted to the zinc release, which is a question of serious debate amongst organizations in Europe [58]. The studies of Smolders and Degryse [20] and Councell et al. [22] suggest that Zn in rubber tires may be mobile and bioavailable; elevated Zn levels have been demonstrated in leaching experiments using tire rubber [29] and simulated rainwater [34,59]. Zn leaching can be augmented indirectly by changing local ambient conditions (e.g., by lowering pH) and it has been demonstrated that tire wear eluates are toxic to species of alga, Daphnia and fish [29,60]. This toxicity is related to the presence of zinc in the eluates, and the zinc eluted concentration is always higher than that considered harmful to aquatic organisms. Actually maximum permissible Zn concentrations in water, based on toxicity to aquatic organisms, have been set by EPA at 120 μg/l.

Eluates obtained with 50 and 100 g/L TD at a dilution of 50% and undiluted produced high toxicity in *X. laevis *embryos. Luo et al.[49] showed that zinc chloride LC_50 _in FETAX was 55 μg/ml. This concentration produced embryotoxicity and teratogenicity, and it is comparable to that present in our eluates. Our results show that 100 g/L TD eluate is less toxic than 50 g/L. Since TD particles tend to aggregate according to their physical properties, aggregated particles expose to the medium a lower surface area than the dispersed ones. Consequently, physical and chemical interactions between particles and medium are decreased, and less quantity of chemicals is leached in the medium.

It is well documented that zinc can affect living organisms, but its effect on cell lines has received scarce attention. We have exposed the HepG2 cell line to the metal salt ZnSO_4._7H_2_O at different concentrations from 0.05 to 50 μg/ml. The 50 μg/ml dose produced a heavy uptake of the metal, and the ICP-AES analysis revealed that the intracellular level of zinc increased from 4 to 24 h of exposure. The analytical procedure used showed a time-dependent accumulation of zinc in cells exposed for 2, 4 and 24 h, leading to a cytotoxic effect. When the intracellular level of zinc exceeds a certain threshold, a number of homeostatic mechanisms take part in regulating the inside distribution by acting on the flux across the plasma membrane, by sequestering the zinc within subcellular compartments [43], or by synthesising molecules able to bind zinc tightly [61].

When tire eluates were treated with EDTA by Abernethy et al. [42] and Gualtieri et al. [39] only partial removal of zinc toxicity was observed, and this indicated the presence of an additional organic toxicant. In fact, several reports [23,54,55,60] refer to the presence of organic components that can be leached. The TD organic fraction, obtained by extraction in soxhlet apparatus and analysed with FTIR, contained isoprene as the principal chemical compound. Isoprene is an industrial chemical widely used as the basic monomeric unit in natural rubber and the chemical used predominantly in manufacturing of poly-isoprene and various copolymers, such as butyl rubber. Inhalation studies have demonstrated multiple organ tumorigenic effects with this chemical in mice and rats [62,63]. Isoprene belong to the terpens chemical family, and several studies were conducted on these compounds. Isoprene and chloroprene are listed in the National Toxicology Program's Report on Carcinogens as reasonably anticipated to be a human carcinogen, even though epidemiology data about these two chemicals are not considered adequate to evaluate their potential carcinogenicity in humans. Moreover, it has recently been demonstrated that isoprene forms guanidine adducts [64], and that methoprene and its degradation products affect *X. laevis *development [65].

The interaction between inhaled particles and lung cells is described in literature [66,67], as well as the correlation between tire particles and the release of latex allergy proteins [23,68]. These data show that TD can release chemical compounds of its matrix. Recently, it has also been suggested that TD extractable chemicals are endocrine disruptors [69], which justifies the increasing interest in this pollutant.

We exposed both the A549 cell line and *X. laevis *embryos to TD extracts. A549 cell proliferation decreased in a time- and dose-dependent manner, and a significant statistical decrease appeared at 24 h and became more evident at 48 and 72 h at the doses tested. To define the role of TD organic extract as an inducer of damage in cellular DNA, we assessed DNA strand breakage by using a comet assay and found that extracts caused strand break formation. It has been shown using the comet assay that insoluble particles, such as carbon black [8] and quarz [70], were able to induce strand breaks in A549 cells, probably via intracellular generation of OH. Further investigation is needed to determine if this is the mechanism by which TD organic extracts act. Organic TD treatment greatly modified the morphology of these cells, and apoptotic figures in the nucleus and the cytoplasm were frequent at the highest dose. Moreover, TD extracts produced significant toxicity in *X. laevis *embryos, since a significant difference in mortality percentages between the control and the highest dose exposed group was present. Moreover an increase of malformed larvae percentages was evident.

## 5.Conclusions

Previous work and these results confirm the significant role of zinc in leached TD and the presence of additional organic toxicants. The studies performed have focused their attention on the potential toxic risk to living aquatic organisms from whole rubber tires or scrap. In this study TD has been investigated for its impact on human cell lines and on *X. laevis *embryos. TD eluates contain zinc, and we have demonstrated that this metal can accumulate in cells, and affect *X. laevis *embryos. The TD organic extract was toxic to A549 cells and affected cell morphology, cell proliferation and DNA, and produced severe malformations in developing *X. laevis *embryos. These results contribute to the knowledge of a PM component, which represents a considerable PM_10 _percentage, which at present is not considered a 'hazardous substance", but it must be taken into account for its potential environmental impact [71,33]. Moreover, these results strongly stress the need for further investigation into the distribution of TD as well as on its fate in urban areas.

## Abbreviations

Tire Debris = TD; Particulate matter = PM; Frog Embryo Teratogenesis Assay-*Xenopus *= FETAX; Diesel Exhaust Particles = DEP; Polycyclic Aromatic Hydrocarbon = PAH; Focused Ion Beam = FIB; Transmission Electron Microscopy = TEM; Scanning Electron Microscopy = SEM; Inductively Coupled Plasma-Atomic Emission Spectrometry = ICP-AES; Dimethyl Sulfoxide = DMSO; 3-(4,5-dimethylthiazol-2-yl)-2,5-diphenyltetrazolium bromide = MTT; Human Chorionic Gonadotropin = HCG; Fourier Transformed InfraRed analysis = FTIR

## Competing interests

The author(s) declare that they have no competing interests.

## Authors' contributions

MG conceived of the study, carried out the proliferation and other biochemical assays on cell lines, drafted the manuscript. MA carried out the FETAX test and drafted the manuscript. PM drafted the manuscript. CV performed the statistical analysis. MC conceived of the study and participated in its design and coordination and helped to draft the manuscript. All authors read and approved the final manuscript.
